# ^68^Ga–Prostate-Specific Membrane Antigen–Avid Malignant Pleural Effusion in a Patient With Metastatic Adenoid Cystic Carcinoma and Concordance With ^18^F-FDG PET/CT

**DOI:** 10.1097/RLU.0000000000004015

**Published:** 2022-01-07

**Authors:** Maike José Maria Uijen, Jetty Anne Mina Weijers, Chantal Maria Leonarda Driessen, Carla Marie Louise van Herpen, James Nagarajah

**Affiliations:** From the ∗ Department of Medical Oncology, Radboud Institute for Health Sciences; † Department of Medical Imaging, Nuclear Medicine, Radboud Institute for Molecular Life Sciences, Radboud University Medical Center, Nijmegen, the Netherlands.

**Keywords:** adenoid cystic carcinoma, malignant pleural effusion, ^68^Ga-PSMA-11 PET/CT, ^18^F-FDG PET/CT

## Abstract

Adenoid cystic carcinoma (ACC) is a rare cancer that arises from salivary glands and other secretory glands. Pulmonary metastases are frequently observed in ACC patients with metastatic disease. Previous research showed that ACC often shows high PSMA uptake on ^68^Ga-PSMA-11 PET/CT. Here, we present PET images from an ACC patient with pulmonal, pleural metastases, and malignant pleural effusion, with comparable tracer uptake on ^68^Ga-PSMA-11 PET and ^18^F-FDG PET.

**FIGURE 1:**
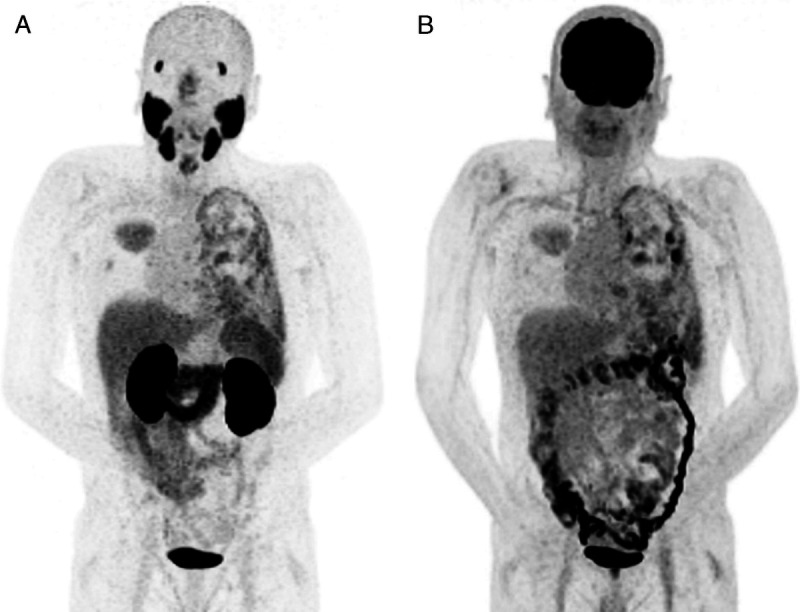
A 59-year-old man with a history of adenoid cystic carcinoma (ACC) from the nasopharynx has previously been treated with surgery and postoperative radiotherapy. Eight years after the primary treatment, pulmonary metastases were diagnosed on a chest radiograph, which was performed because of a persisting cough. In a subgroup of patients with ACC disease, distant metastases can be detected several years after initial diagnosis, often related due to the indolent tumor growth of ACC and often asymptomatic metastases.^1,2^ In case of recurrent or metastatic ACC disease, systemic therapy should mainly be considered for patients with symptomatic disease or rapid progression.^3,4^ Although there is still no standard systemic treatment for ACC, previous single-arm phase 2 studies showed the efficacy of combinations of cytotoxic drugs^5^ or a tyrosine kinase inhibitor.^6,7^ Yet, response rates of these drugs are moderate, and participation in clinical trials is advised.^4^ Previously, because of symptomatic disease, the patient received cabozantinib treatment in a clinical trial. Unfortunately, the clinical trial closed prematurely due to severe toxicity in other included patients, and the cabozantinib treatment was discontinued after 1 month.^8^ After 1 year, his disease-related symptoms increased, and recently, the patient developed malignant pleural effusion (diagnosed on imaging, not validated with pleural puncture), without dyspnea. Therefore, other systemic treatment options were considered. Recently, this patient received both ^68^Ga-PSMA-11 PET and ^18^F-FDG PET imaging as part of the screening procedure for a ^177^Lu-PSMA radioligand therapy study. Because ACC tumors are often avid on ^68^Ga-PSMA PET/CT imaging^9^ and PSMA radioligand therapy has shown good results in prostate cancer,^10,11^ we initiated a clinical trial (NCT04291300) applying ^177^Lu-PSMA radioligand therapy for salivary gland cancer patients, including ACC patients. The time interval between the ^68^Ga-PSMA-11 PET and ^18^F-FDG PET scan was <1 week. In addition to a comparable uptake on both PET scans of the pulmonal and pleural metastases, the malignant pleural effusion was also clearly visible. PSMA PET imaging is most often performed in prostate cancer patients, yet malignant pleural effusion in prostate cancer is rare and only 1 case report on ^68^Ga-PSMA–avid malignant pleural effusion in prostate cancer has been reported.^12^ PSMA PET imaging is lately being investigated for several cancers other than prostate cancer.^13^ **A**, Image shows the MIP image of the ^68^Ga-PSMA-11 PET. **B**, Image shows the MIP image of the ^18^F-FDG PET.

## References

[1] Spiro RH. Distant metastasis in adenoid cystic carcinoma of salivary origin. *Am J Surg*. 1997;174:495–498. 9374223 10.1016/s0002-9610(97)00153-0

[2] Sung MW Kim KH Kim JW, . Clinicopathologic predictors and impact of distant metastasis from adenoid cystic carcinoma of the head and neck. *Arch Otolaryngol Head Neck Surg*. 2003;129:1193–1197. 14623749 10.1001/archotol.129.11.1193

[3] Geiger JL Ismaila N Beadle B, . Management of salivary gland malignancy: ASCO guideline. *J Clin Oncol*. 2021;39:1909–1941. 33900808 10.1200/JCO.21.00449

[4] Laurie SA Ho AL Fury MG, . Systemic therapy in the management of metastatic or locally recurrent adenoid cystic carcinoma of the salivary glands: a systematic review. *Lancet Oncol*. 2011;12:815–824. 21147032 10.1016/S1470-2045(10)70245-X

[5] Licitra L Cavina R Grandi C, . Cisplatin, doxorubicin and cyclophosphamide in advanced salivary gland carcinoma. A phase II trial of 22 patients. *Ann Oncol*. 1996;7:640–642. 8879381 10.1093/oxfordjournals.annonc.a010684

[6] Tchekmedyian V Sherman EJ Dunn L, . Phase II study of lenvatinib in patients with progressive, recurrent or metastatic adenoid cystic carcinoma. *J Clin Oncol*. 2019;37:1529–1537. 30939095 10.1200/JCO.18.01859 PMC6599407

[7] Zhu G Zhang L Li R, . Phase II trial of apatinib in patients with recurrent and/or metastatic adenoid cystic carcinoma of the head and neck: updated analysis. *J Clin Oncol*. 2018;36:6026–6026.

[8] Van Boxtel W Uijen M Driessen C, . A phase II study on the efficacy and toxicity of cabozantinib in recurrent/metastatic salivary gland cancer patients. *J Clin Oncol*. 2020;38(suppl 15):6529–6529.

[9] van Boxtel W Lütje S van Engen-van Grunsven ICH, . ^68^Ga-PSMA-HBED-CC PET/CT imaging for adenoid cystic carcinoma and salivary duct carcinoma: a phase 2 imaging study. *Theranostics*. 2020;10:2273–2283. 32089741 10.7150/thno.38501 PMC7019174

[10] Hofman MS Violet J Hicks RJ, . [(177)Lu]-PSMA-617 radionuclide treatment in patients with metastatic castration-resistant prostate cancer (LuPSMA trial): a single-centre, single-arm, phase 2 study. *Lancet Oncol*. 2018;19:825–833. 29752180 10.1016/S1470-2045(18)30198-0

[11] Sartor O de Bono J Chi KN, . Lutetium-177–PSMA-617 for metastatic castration-resistant prostate cancer. *N Engl J Med*. 2021;385:1091–1103. 34161051 10.1056/NEJMoa2107322 PMC8446332

[12] Sachpekidis C Alberts I Rominger A, . ^68^Ga-prostate-specific membrane antigen uptake in a malignant pleural effusion from metastatic prostate cancer after pleurodesis. *Clin Nucl Med*. 2019;44:838–839. 31283614 10.1097/RLU.0000000000002748

[13] Uijen MJM Derks YHW Merkx RIJ, . PSMA radioligand therapy for solid tumors other than prostate cancer: background, opportunities, challenges, and first clinical reports. *Eur J Nucl Med Mol Imaging*. 2021;48:4350–4368. 34120192 10.1007/s00259-021-05433-w PMC8566635

